# Early Changes in Pain Acceptance Predict Pain Outcomes in Interdisciplinary Treatment for Chronic Pain

**DOI:** 10.3390/jcm8091373

**Published:** 2019-09-02

**Authors:** Thomas Probst, Robert Jank, Nele Dreyer, Stefanie Seel, Ruth Wagner, Klaus Hanshans, Renate Reyersbach, Andreas Mühlberger, Claas Lahmann, Christoph Pieh

**Affiliations:** 1 Department for Psychotherapy and Biopsychosocial Health, Danube University Krems, 3500 Krems, Austria; 2 Institute for Psychology, Regensburg University, 93053 Regensburg, Germany; 3 Hospital Barmherzige Brüder, 93049 Regensburg, Germany; 4 Department of Psychosomatic Medicine and Psychotherapy, University Medical Center Freiburg, Faculty of Medicine, University of Freiburg, 79106 Freiburg, Germany

**Keywords:** chronic pain, pain acceptance, early change, interdisciplinary pain treatment

## Abstract

Studies have shown that pain acceptance is associated with a better pain outcome. The current study explored whether changes in pain acceptance in the very early treatment phase of an interdisciplinary cognitive-behavioral therapy (CBT)-based treatment program for chronic pain predict pain outcomes. A total of 69 patients with chronic, non-malignant pain (at least 6 months) were treated in a day-clinic for four-weeks. Pain acceptance was measured with the Chronic Pain Acceptance Questionnaire (CPAQ), pain outcomes included pain intensity (Numeric Rating Scale, NRS) as well as affective and sensory pain perception (Pain Perception Scale, SES-A and SES-S). Regression analyses controlling for the pre-treatment values of the pain outcomes, age, and gender were performed. Early changes in pain acceptance predicted pain intensity at post-treatment measured with the NRS (B = −0.04 (SE = 0.02); T = −2.28; *p* = 0.026), affective pain perception at post-treatment assessed with the SES-A (B = −0.26 (SE = 0.10); T = −2.79; *p* = 0.007), and sensory pain perception at post-treatment measured with the SES-S (B = −0.19 (SE = 0.08); T = −2.44; *p* = 0.017). Yet, a binary logistic regression analysis revealed that early changes in pain acceptance did not predict clinically relevant pre-post changes in pain intensity (at least 2 points on the NRS). Early changes in pain acceptance were associated with pain outcomes, however, the impact was beneath the threshold defined as clinically relevant.

## 1. Introduction

Chronic pain (CP) is a serious clinical problem [1]. In the United States, more than 100 million people suffer from CP and the annual costs for the society range between $560 and $635 billion [2]. As the presence of pain affects all aspects of an individual’s functioning, an interdisciplinary approach incorporating the knowledge and skills of different healthcare providers is essential [3].

Interdisciplinary treatments for CP are based on the bio-psycho-social model of pain [4]. Such interdisciplinary treatments are conducted by a multi-professional team and address biological factors (e.g., medication, exercise), psychological factors (e.g., cognitions, emotions), as well as social factors (e.g., family, work) [4]. Several studies on CP showed that such interdisciplinary interventions are efficacious in randomized controlled trials [5,6] as well as effective under the conditions of routine care [7,8]. 

Psychotherapy is a central component in interdisciplinary pain treatments and cognitive-behavioral therapy (CBT) is the most often used [9]. Several meta-analyses and reviews have evaluated the efficacy of CBT for patients with CP [10]. A Cochrane review [11] revealed that CBT had statistically significant but small effects on pain and disability. The effects on mood and catastrophizing were moderate. These effects were compared with treatment-as-usual and wait-list control conditions. An integrative review of Knoerl et al. reported that CBT reduced pain intensity in 43% of the trials [12].

Another psychotherapeutic approach for the treatment of CP is Acceptance and Commitment Therapy (ACT; [13]). Acceptance and Commitment Therapy is a contextual form of CBT. Recent reviews reported that ACT is beneficial for patients with CP [14,15]. The ACT aims at increasing psychological flexibility (PF) and decreasing its counterpart psychological inflexibility [13,16]. While psychological inflexibility is associated with psychological problems or even psychiatric symptoms, PF is defined as the capacity to be in conscious and open contact with one’s thoughts and feelings, and to behave according to one’s values and goals [17]. Psychological flexibility consists of six core components (acceptance, cognitive defusion, self as a context, committed action, values, contact with the present moment) [18]. In the context of CP, pain acceptance is central to PF. Pain acceptance refers to the degree to which a patient is willing to live with pain or decides to get on with life despite pain. The Chronic Pain Acceptance Questionnaire (CPAQ; [19]) is a psychometrically sound instrument to operationalize pain acceptance and consists of a total scale and the following two subscales: Activity engagement and pain willingness. Activity engagement refers to the performance of personally valued activities, even in the presence of pain. Pain willingness refers to the willingness to give up attempts to control or avoid pain. Several studies on ACT for CP found that improvements in pain acceptance are associated with better pain outcomes [20,21,22]. Yet, two other studies suggest that pain acceptance is a change mechanism in other treatments as well and no ACT specific treatment process. These studies investigated whether improvements in pain acceptance are correlated with pain outcomes in interdisciplinary CBT-based treatments. Baranoff et al. [23] reported that pre-post improvements in pain acceptance were associated with improvements in almost all outcomes at the end of an interdisciplinary CBT-based treatment for CP. In another study on interdisciplinary CBT-based treatment for CP, Akerblom et al. [9] found that pain acceptance was not related to the outcome pain intensity but that it was the strongest mediator for the other outcome measures. These studies showed that improvements in pain acceptance play a crucial role for pain outcomes in different therapeutic approaches for CP. Baranoff et al. as well as Akerblom et al. assessed pain acceptance at pre-treatment, post-treatment, and at follow-up, but not during the interdisciplinary CBT-based treatment. To extend these previous findings, pain acceptance was monitored during interdisciplinary CBT-based treatment for CP in this project. The aim of the current study was to evaluate whether changes of pain acceptance in the very early treatment phase (first week) predict pain outcomes at post-treatment. We hypothesized that such early changes related to PF are predictors of pain outcomes at post-treatment, since early changes of variables related to psychological inflexibility (e.g., catastrophizing, depression, anxiety) have already been shown to be predictors of changes in pain and interference [24,25,26,27].

## 2. Materials and Method

### 2.1. Measurements

#### 2.1.1. Pain Acceptance

Pain acceptance was measured with the Chronic Pain Acceptance Questionnaire (CPAQ [19,28]). The CPAQ consists of 20 items and has satisfactorily psychometric properties [19,28,29]. For the present study, only the total score of the CPAQ was analyzed. In the sample of the current study, the internal consistency at pre-treatment was Cronbachs Alpha α = 0.87. Higher CPAQ scores indicate higher pain acceptance. Early changes in pain acceptance were operationalized as the difference between the CPAQ scores after the first treatment week and the CPAQ scores at the beginning of treatment. Differences were calculated so that more positive differences indicate more improvements in pain acceptance.

#### 2.1.2. Pain Outcomes

Pain was measured with (1) an 11-point Numerical Rating Scale (NRS) and (2) the Pain Perception Scale. The NRS is a common self-rating instrument to measure pain intensity in patients with CP from 0 (no pain) to 10 (worst imaginable pain). The psychometric properties of the NRS have been investigated in several studies [30,31]. In the current study, self-ratings on average pain intensity were investigated. The Pain Perception Scale (SES) has 28 items and measures affective and sensory pain perception [32]. Both scales showed good internal consistencies in our sample at pre-treatment, with Cronbachs Alpha α = 0.93 for the affective pain perception scale (SES-A) and Cronbachs α = 0.88 for the sensory pain perception scale (SES-S). The pre-treatment and post-treatment scores of the NRS and SES were analyzed in the current study.

### 2.2. Study Sample

All patients taking part in the interdisciplinary CBT-based treatment for CP at the Hospital Barmherzige Brüder, Regensburg (Germany) between June 2014 and November 2015 were asked to participate in the study. During this time interval, N = 69 patients with CP were treated in a day-clinic setting for four weeks and all of them gave written informed consent to partake in this study. All patients suffered from chronic, non-malignant pain according to the ICD-10 diagnosis “chronic pain disorder with somatic and psychological factors (F45.41)”. Of the patients, 36.2% had a comorbid depression diagnosis, 50.7% fulfilled the criteria for at least one comorbid psychiatric disorder.

### 2.3. Intervention

The interdisciplinary treatment was performed according to the pain treatment program “Marburger Schmerzbewältigungsprogramm” [33]. It is a CBT-based program for CP. The patients received treatment for four weeks. Each week treatment lasted from 8:00 a.m. to 4:00 p.m. between Monday and Thursday as well as from 08:00 a.m. to 01:15 p.m. at Friday. The treatment consisted of individual treatment as well as group therapy (closed groups; up to 8 patients per group). The interdisciplinary pain treatment was performed by a team of physicians, psychologists, physical therapists, occupational therapists, and social workers. The CBT component focused mainly on psychoeducation, the bio-psycho-social pain model, relaxation training, and directing the attention towards positive experiences in group sessions and individual sessions. The CBT-based group sessions took part four times per week, and the individual sessions once a week.

### 2.4. Statistical Analysis

All analyses were conducted with SPSS25. To evaluate pre-post changes of pain outcomes (NRS; SES-A; SES-S), we conducted paired t-tests and calculated effect sizes (d) according to the following formula: (M_prä_ − M_post_)/SD_prä_. Effect sizes are interpreted as follows: d ≥ 0.20 small effect, d ≥ 0.50 medium effect, d ≥ 0.80 large effect. Linear regression analyses (method selection “enter”) were performed to address the research question whether early changes in pain acceptance predict pain outcomes at post-treatment. One linear regression model was performed for each pain outcome, i.e., either the NRS at post-treatment, or the SES-A at post-treatment, or the SES-S at post-treatment were the dependent variable. As predictors, we added early changes in pain acceptance, the pre-treatment scores of the respective pain outcome variable (to control for pre-treatment values), age, and gender. Moreover, a logistic regression analysis (method selection “enter”) was performed to evaluate whether early changes in pain acceptance during the first treatment week predict clinically relevant changes in pain intensity from pre- to post-treatment (≥2 points improvement on the NRS from pre- to post-treatment) [34]. The dichotomized NRS (0 = pre-post NRS improvement < 2 points; 1 = pre-post NRS improvement ≥2 points) was the dependent variable, and predictors were early changes in pain acceptance, age, and gender. We included age and gender in the regression models, since they have been shown to influence pain outcomes in previous studies [35,36]. Moreover, Pearson correlation coefficients were calculated between the measures at pre-treatment to investigate potential overlap between the measures. The significance level was set at *p* < 0.05 and the statistical tests were performed two-tailed. Missing data were replaced by the average of the time series.

### 2.5. Ethical Consideration

The Ethics Committee of the University Clinic Regensburg approved the materials and methods for this study. All patients gave written informed consent.

## 3. Results

The sample comprised N = 69 patients with CP (49 females), who were on average M = 52.62 (standard deviation (SD) = 9.78) years old. The participants gave written informed consent to participate in the study, but during the study, some patients did not fill in the measures or dropped out from treatment. Table 1 shows how many patients filled in the applied measures.

The correlations between the measures at pre-treatment are presented in Table 2. Pain intensity was positively correlated with affective pain perception (r = 0.56; *p* < 0.001) and with sensory pain perception (r = 0.25; *p* = 0.040). Affective and sensory pain perception were also positively correlated (r = 0.57; *p* < 0.001). Significantly negative correlations emerged between pain intensity and pain acceptance (r = −0.27; *p* = 0.025) as well as between affective pain perception and pain acceptance (r = −0.48; *p* < 0.001).

Results of the analyses evaluating pre-post changes of the pain outcomes are presented in Table 3. Pain intensity improved (t(68) = 5.82; *p* < 0.001) with a large effect size of d = 0.81, affective pain perception improved (t(68) = 4.43; *p* < 0.001) with a medium effect size of d = 0.60, and sensory pain perception improved (t(68) = 3.26; *p* = 0.002) with a small effect size of d = 0.41.

Three linear regression analysis were conducted to evaluate if early changes in pain acceptance predict the pain outcomes (NRS, SES-A, SES-S) at post-treatment. As can be seen in Table 4, early changes in pain acceptance were negatively correlated with the three pain outcomes. This means that early improvements in pain acceptance were associated with more favorable pain outcomes (i.e., less pain intensity, less affective pain perception, and less sensory pain perception at post-treatment), since higher values on the NRS, SES-A, and SES-S indicate more severe pain, whereas more positive CPAQ difference scores indicate larger early improvements in pain acceptance.

Outcome pain intensity (NRS): R^2^ was 0.17 (F(4, 64) = 3.34, *p* = 0.015). Early changes in pain acceptance predicted pain intensity at post-treatment (B = −0.04 (standard error (SE) = 0.02); T = −2.28; *p* = 0.026) when statistically controlling for pain intensity at pre-treatment (B = 0.34 (SE = 0.12); T = 2.81; *p* = 0.007), age (B = −0.01 (SE = 0.02); T = −0.46; *p* = 0.648), and gender (B = 0.07 (SE = 0.43); T = 0.17; *p* = 0.869). 

Outcome affective pain perception (SES-A): R^2^ reached 0.31 (F(4, 64) = 7.27, *p* < 0.001). Affective pain perception at post-treatment was predicted by early changes in pain acceptance (B = −0.26 (SE = 0.10); T = −2.79; *p* = 0.007) when statistically controlling for affective pain perception at pre-treatment (B = 0.41 (SE = 0.11); T = 3.66; *p* = 0.001), age (B = −0.17 (SE = 0.11); T = −1.48; *p* = 0.145), and gender (B = 1.74 (SE = 2.43); T = 0.72; *p* = 0.476).

Outcome sensory pain perception (SES-S): R^2^ amounted to 0.27 (F(4, 64) = 5.91, *p* < 0.001). When statistically controlling for sensory pain perception at pre-treatment (B = 0.35 (SE = 0.10); T = 3.50; *p* = 0.001), age (B = −0.10 (SE = 0.09); T = −1.10; *p* = 0.276), and gender (B = −0.53 (SE = 1.95); T = −0.27; *p* = 0.787), sensory pain perception at post-treatment was predicted by early changes in pain acceptance (B = −0.19 (SE = 0.08); T = −2.44; *p* = 0.017).

For the outcome pain intensity, we calculated how many patients show a clinically relevant improvement from pre- to post-treatment of at least 2 points on the NRS [34]. N = 31 (45%) improved at least 2 points from pre- to post-treatment and N = 38 (55%) did not. A logistic regression analysis was performed to investigate whether early changes in pain acceptance predict clinically relevant pre-post changes in pain intensity. The results of this logistic regression (−2 Log-Likelihood = 90.77; Cox and Snell R-Quadrat = 0.06; Nagelkerkes R-Quadrat = 0.08) showed that early changes in pain acceptance do not predict clinically significant NRS changes (Exp(B) = 1.04; 95% confidence interval (CI): 0.99; 1.09; *p* = 0.157) when controlling for age (Exp(B) = 1.03; 95% CI: 0.98; 1.09; *p* = 0.304) and gender (Exp(B) = 1.07; 95% CI: 0.36; 3.25; *p* = 0.900).

## 4. Discussion

This study evaluated whether early changes in pain acceptance predict pain outcomes at the end of an interdisciplinary CBT-based treatment for CP. As pain outcomes, we evaluated pain intensity, affective pain perception, and sensory pain perception. The results showed that early changes in pain acceptance within the first treatment week were associated with less pain intensity, less affective pain perception, and less sensory pain perception at the end of an interdisciplinary pain program. However, clinically relevant changes in pain intensity from pre- to post-treatment were not predicted by early changes in pain acceptance.

Our results extend past research on pain acceptance in interdisciplinary CBT-based treatments for CP [9,23], which showed that changes in pain acceptance are associated with outcomes. Yet, the previous studies did not investigate pain acceptance in the early treatment phase. With regard to the pain outcome pain intensity, our results appear to be in contrast to another study [9] where the outcome pain intensity was not correlated with changes in pain acceptance. One explanation could be that Akerblom et al. [9] analyzed pre-post changes in pain acceptance, whereas we investigated early changes in pain acceptance during the first treatment week. It might be that the outcome pain intensity is predicted by early changes in pain acceptance but not by pre-post changes in pain acceptance. This speculation receives some support from pain studies showing that different predictors of the outcome can be found in the early and late treatment phase [24,25,26,27].

When interpreting our results, it should be kept in mind that pain acceptance is no explicit treatment target in CBT-based treatments. We can only speculate about the factors influencing early changes in pain acceptance. Possibly, non-specific common factors like the therapeutic alliance or hope might be associated with early changes in pain acceptance as these common factors have been discussed to play a role in the early phase of psychotherapy [37,38], but this needs to be further studied in the area of pain. It should also be kept in mind that some of the pain outcome measures (NRS, SES-A) were significantly correlated with the pain acceptance measure at pre-treatment. Therefore, the correlation between changes in pain acceptance and changes in pain intensity as well as changes in affective pain perception may be overstated. There are several further shortcomings to discuss. Although the external validity/generalizability of the results is positively influenced by the conditions of routine practice, the following points limit the generalizability. All patients had the ICD-10 diagnosis “chronic pain disorder with somatic and psychological factors (F45.41)” and it remains unclear how early changes in pain acceptance influence pain outcomes in patients with other forms of pain. The representativeness is further reduced by the rather small sample size (N = 69) as well as the relatively large amount of missing data. The sample is, for example, too small for a sound investigation of moderators between early changes in pain acceptance and pain outcomes. Future larger studies could include age and gender as moderators and investigate whether the effect of early changes in pain acceptance on pain outcomes interacts with age and gender. Besides CP, more than half of the patients had various, especially psychiatric diagnoses. Another limitation is that the diagnoses were made by the clinic team, but not with a structured or standardized clinical interview. Due to the naturalistic design we did not calculate a power analysis ahead of the study. We replaced missing data by the average of the time series. This approach leads to an overestimation of the effect compared to replacing missing post-treatment data with the pre-treatment values. Other strategies to handle missing data (e.g., Expectation-Maximization algorithm) might lead to different results. Furthermore, regression analysis does not allow drawing causal inferences and the internal validity of the results is rather low. A randomized controlled trial comparing a condition including a component to increase pain acceptance in the early treatment phase and a condition excluding this component would produce results of higher internal validity. Based on our results, one would expect that pain outcomes are better in the condition with the component to increase pain acceptance in the early treatment phase. A further limitation is that we only included pain acceptance as process variable. The inclusion of more process variables (e.g., pain catastrophizing) would have allowed to investigate whether early changes in one process variable are more or less important predictors of pain outcomes than early changes in other process variables. Another shortcoming is that pain intensity and pain perception were the solely outcomes in the current study and other outcomes such as functioning or patient satisfaction should also be integrated to evaluate treatments for CP [39]. Pain acceptance might also be an important outcome in treatments for CP and we analyzed pain acceptance as a process measure only. Furthermore, our results rely on self-report data and complementary assessments of more objective pain outcomes (e.g., quantitative sensory testing) would be welcome in future studies. Moreover, our definition of early change should be discussed in more detail. We investigated difference scores within the first week to investigate early changes in pain acceptance. The first treatment week might a suitable time frame to study early changes, but other studies defined the change from pre- to mid-treatment [25,26], or the change from pre-treatment to the third treatment week [24,40] as early change. It should also be mentioned that there are several other approaches to operationalize change rate [41] such as deviations from expected recovery curves [40], sudden gains [42] reliable change [43] or the method of percent of improvement [44]. Finally, we only investigated how early changes in pain acceptance influence the short-term outcome at the end of the interdisciplinary treatment for CP, but we do not know how the long-term outcome is predicted by early changes of pain acceptance due to the lack of follow-up assessments.

## 5. Conclusions

Early changes in pain acceptance are associated with continuous pain outcomes, but not with clinically relevant improvements in pain intensity.

## Figures and Tables

**Table 1:** Number of patients filling in the questionnaires. N = 69.

Measure	Number of Patients Filling in the Measure N (%)
NRS pre-treatment	66 (96%)
NRS post-treatment	53 (77%)
SES-A pre-treatment	61 (88%)
SES-A post-treatment	52 (75%)
SES-S pre-treatment	62 (90%)
SES-S post-treatment	50 (72%)
CPAQ pre-treatment	52 (75%)
CPAQ after first treatment week	61 (88%)

Note: NRS = Numeric Rating Scale; SES-A = Affective Pain Perception Scale; SES-S = Sensory Pain Perception Scale; CPAQ = Chronic Pain Acceptance Questionnaire.

**Table 2:** Correlations between the measures at pre-treatment. N = 69.

		SES-A	SES-S	CPAQ
NRS	r	0.56	0.25	−0.27
	*p*-value	<0.001	0.040	0.025
SES-A	r		0.57	−0.48
	*p*-value		<0.001	<0.001
SES-S	r			−0.23
	*p*-value			0.061

Note: NRS = Numeric Rating Scale; SES-A = Affective Pain Perception Scale; SES-S = Sensory Pain Perception Scale.

**Table 3:** Pre–post changes in pain outcomes. N = 69.

	Pre-Treatment M (SD)	Post-Treatment M (SD)	Statistics	Effect Size
Pain intensity (NRS)	6.33 (1.62)	4.97 (1.65)	t(68) = 5.82; *p* < 0.001	d = 0.84
Affective Pain Perception (SES-A)	35.90 (9.60)	30.12 (10.21)	t(68) = 4.43; *p* < 0.001	d = 0.60
Sensory Pain Perception (SES-S)	31.94 (8.64)	28.38 (8.02)	t(68) = 3.26; *p* = 0.002	d = 0.41

Note: NRS = Numeric Rating Scale; SES-A = Affective Pain Perception Scale; SES-S = Sensory Pain Perception Scale; SD = standard deviation.

**Table 4:** Results of the multiple regression analyses testing early changes in pain acceptance as predictors of pain outcomes. N = 69.

	Unstandardized Coefficients	Standardized Coefficients		
	B (SE)	Beta	T	*p*-Value
***Pain intensity (NRS)***					
(Constant)	3.31 (1.21)		2.74	0.008
Early changes in pain acceptance (CPAQ)	−0.04 (0.02)	−0.27	−2.28	0.026
NRS pre-treatment	0.34 (0.12)	0.33	2.81	0.007
Age	−0.01 (0.02)	−0.06	−0.46	0.648
Gender	0.07 (0.43)	0.02	0.17	0.869
***Affective Pain Perception (SES-A)***				
(Constant)	22.45 (7.03)		3.20	0.002
Early changes in pain acceptance (CPAQ)	−0.26 (0.10)	−0.31	−2.79	0.007
SES-A pre-treatment	0.41 (0.11)	0.38	3.66	0.001
Age	−0.17 (0.11)	−0.16	−1.48	0.145
Gender	1.74 (2.43)	0.08	0.72	0.476
***Sensory Pain Perception (SES-S)***				
(Constant)	23.53 (6.21)		3.79	<0.001
Early changes in pain acceptance (CPAQ)	−0.19 (0.08)	−0.28	−2.44	0.017
SES-S pre-treatment	0.35 (0.10)	0.38	3.50	0.001
Age	−0.10 (0.09)	−0.12	−1.10	0.276
Gender	−0.53 (1.95)	−0.03	−0.27	0.787

Note: NRS = Numeric Rating Scale; SES-A = Affective Pain Perception Scale; SES-S = Sensory Pain Perception Scale; CPAQ = Chronic Pain Acceptance Questionnaire; SE = standard error.
